# Mutual regulation between N6-methyladenosine (m6A) modification and circular RNAs in cancer: impacts on therapeutic resistance

**DOI:** 10.1186/s12943-022-01620-x

**Published:** 2022-07-18

**Authors:** Hong Lin, Yuxi Wang, Pinghan Wang, Fangyi Long, Ting Wang

**Affiliations:** 1 Department of Pharmacy, Sichuan Cancer Hospital & Institution, Sichuan Cancer Center, School of Medicine, University of Electronic Science and Technology of China, Chengdu, China; 2 Targeted Tracer Research and Development Laboratory, Institute of Respiratory Health, Frontiers Science Center for Disease-related Molecular Network, West China Hospital, Sichuan University, Chengdu, China; 3 Laboratory Medicine Center, Sichuan Provincial Maternity and Child Health Care Hospital, Affiliated Women’s and Children’s Hospital of Chengdu Medical College, Chengdu Medical College, Chengdu, China

**Keywords:** N6-methyladenosine, Circular RNA, Interplay, Cancer, Therapeutic resistance

## Abstract

The resistance of tumor cells to therapy severely impairs the efficacy of treatment, leading to recurrence and metastasis of various cancers. Clarifying the underlying mechanisms of therapeutic resistance may provide new strategies for overcoming cancer resistance. N6-methyladenosine (m6A) is the most prevalent RNA modification in eukaryotes, and is involved in the regulation of RNA splicing, translation, transport, degradation, stability and processing, thus affecting several physiological processes and cancer progression. As a novel type of multifunctional non-coding RNAs (ncRNAs), circular RNAs (circRNAs) have been demonstrated to play vital roles in anticancer therapy. Currently, accumulating studies have revealed the mutual regulation of m6A modification and circRNAs, and their interaction can further influence the sensitivity of cancer treatment. In this review, we mainly summarized the recent advances of m6A modification and circRNAs in the modulation of cancer therapeutic resistance, as well as their interplay and potential mechanisms, providing promising insights and future directions in reversal of therapeutic resistance in cancer.

## Introduction

Cancer is one of the tremendous challenges to human health globally and the leading cause of death [1]. Despite great advances in cancer treatment have significantly reduced the mortality rate, such as the development of novel targeted therapy and immunotherapy, many cancer patients haven’t achieved satisfactory therapeutic outcomes due to the emergence of treatment resistance, and still maintain poor prognosis and high mortality [2]. Therefore, exploring the potential mechanisms that affect cancer therapeutic efficiency is of great significance.

**Fig. 1:**
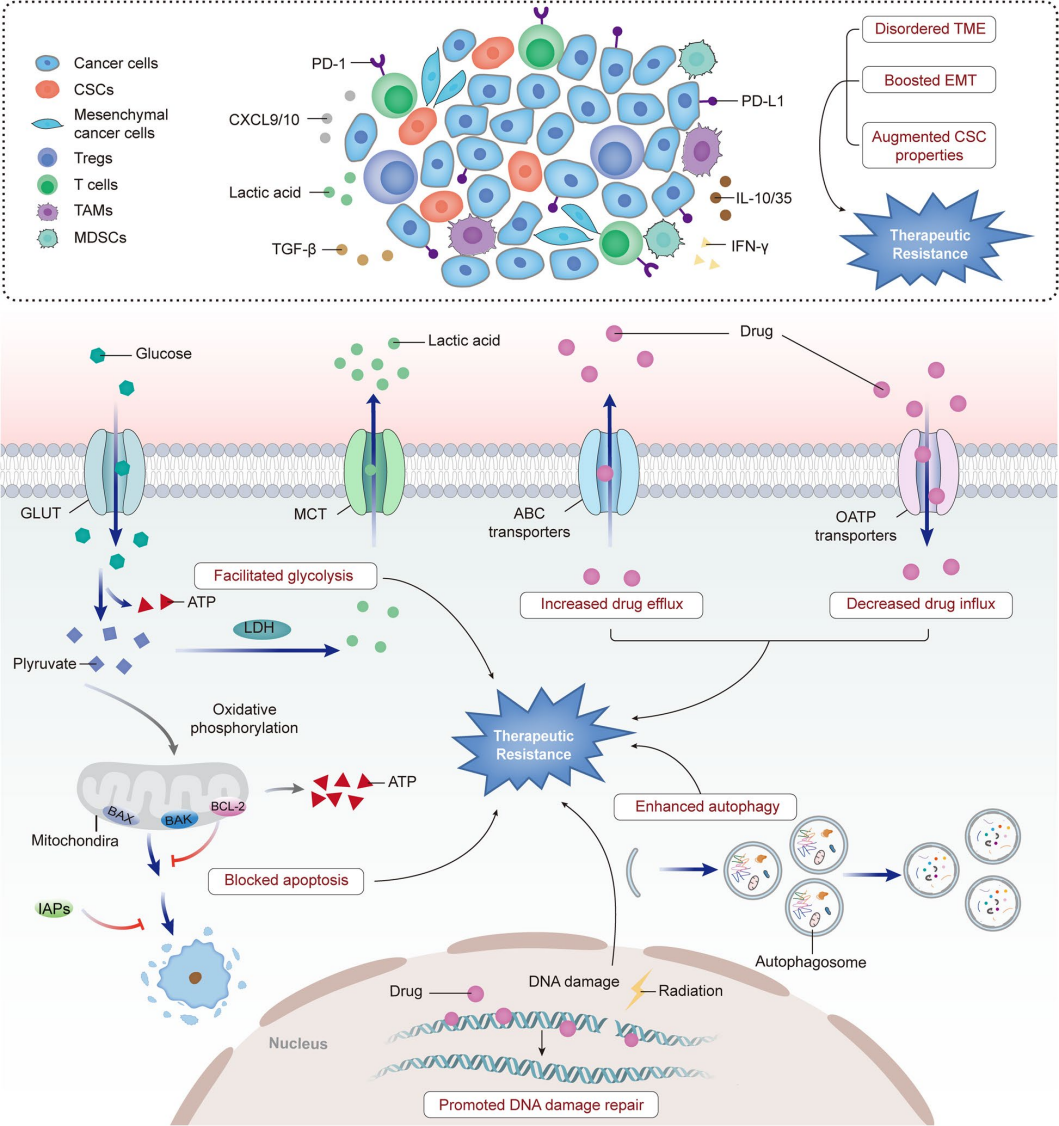
Mechanisms involved in therapeutic resistance in cancer. Increased drug efflux and decreased drug influx lead to reduced accumulation of anticancer drugs in tumor cells. Additionally, blocking apoptosis, promoting DNA damage repair and enhancing autophagy also help tumor cells easily survive under treatment stress. Moreover, tumor cells preferentially consume glucose, producing lactic acid and ATP through glycolysis, contributing to cell rapid proliferation and resistance to therapy. Finally, a disordered tumor microenvironment (TME), promotion of epithelial-mesenchymal transition (EMT) and augmented cancer stem cell (CSC) properties will also restrict the therapeutic efficacy of cancer

Under treatment stress, tumor cells can escape from death, resulting in therapeutic resistance. On one hand, the efficacy of various anticancer drugs could be weakened by alterations in drug transport, metabolism and drug-target interactions. On the other hand, tumor cells themselves could gain survival advantages through mechanisms including anti-apoptosis, DNA damage repair, autophagy activation, remodeling tumor microenvironment (TME), induction of epithelial-mesenchymal transition (EMT), and so on (Fig. 1) [3–5]. Besides, the aberrant expression of non-coding RNAs (ncRNAs) has also been recognized as an essential regulator of cancer chemoresistance and radioresistance. NcRNAs are RNAs molecules that generally lack protein-coding potential, while they could perform distinct biological functions including modulation of therapeutic efficacy in cancer. In recent decades, a large number of ncRNAs have been identified and classified into different subtypes, mainly including microRNAs (miRNAs), long non-coding RNAs (lncRNAs) and circular RNAs (circRNAs) [6]. As a novel type of ncRNA with a covalently closed structure, circRNAs were first discovered in 1976 and identified to participate in regulating the progression of various chronic diseases including cancer [7]. More recently, the roles of circRNAs in cancer therapeutic resistance are increasingly attracting the worldwide attention. For instance, upregulation of hsa_circ_0074298 were revealed to increase secreted modular calcium-binding protein 2 (SMOC2) expression via sponging miR-519, resulting in gemcitabine resistance in pancreatic cancer (PC) [8]. Strikingly, circRNAs are characterized by high stability due to their special closed structures, and an increasing number of circRNAs have been found in human tissues and body fluids including urine, saliva and plasma with tissue- and stage-specific expression, implying that circRNAs are more desirable biomarkers in cancer [9, 10].

RNA modifications play critical roles in regulating gene expression and functions. As the most common RNA modification among over 100 identified types of RNA post-transcriptional modifications, N6-methyladenosine (m6A) modification is involved in important biological processes such as RNA splicing, translation and degradation, and is closely associated with a variety of human diseases, particularly cancer [11]. Abnormal alterations of m6A modification in tumor cells can result in dysregulation of gene expression, including ATP-binding cassette (ABC) transporter gene family, DNA damage and repair genes, anti-apoptotic and pro-apoptotic genes, and contribute to cancer recurrence and therapeutic resistance [12–14]. A recent example is that METTL3-mediated m6A modification promoted the expression of multidrug transporter ABCC1, thereby conferring imatinib resistance in gastrointestinal stromal tumors [15]. Furthermore, growing evidence suggests that m6A modification is also present in ncRNAs including circRNAs, which can modulate its functions in cancer. Interestingly, circRNAs can in turn regulate m6A modification and further affect the expression of target mRNAs, which bring totally new insights into cancer therapeutic resistance [16, 17].

In this review, we thoroughly summarized the research progress and potential mechanisms of m6A modification and circRNAs-related cancer treatment resistance in cancer, as well as their interplay in the regulation of therapeutic resistance, indicating that targeting m6A modification and circRNAs is expected to achieve the goal of reversal of therapeutic resistance in cancer.

## M6A modification and circRNAs

### Important m6A modification regulators

M6A modification, referring to the methylation of the sixth nitrogen (N) atom of RNA adenylate (A), is dynamically regulated by three important regulators consisting of m6A methyltransferases, m6A demethylases and m6A-binding proteins. M6A modification plays important roles in gene expression, thereby influencing many physiological and pathological processes such as cells differentiation, self-renewal and apoptosis, as well as the development of cancer, cardiovascular and metabolic diseases [18–20].

M6A methyltransferases, also known as ‘m6A writers’, are involved in the composition of methyltransferase complex (MTC) to add m6A sites to RNA [21]. MTC is mainly composed of two methyltransferase-like proteins (METTL3/14) and Wilm’s tumor 1-associated protein (WTAP) [22]. METTL3, identified by Joseph et al. in 1997, is the most significant component protein in the complex, with the ability to catalyze the formation of m6A [23]. METTL14 has no catalytic activity, however, it can help METTL3 to recognize substrates by binding to METTL3 to form a heterodimer [24]. WTAP serves as a key component that recruits MTC to RNA targets [25]. Moreover, other m6A methyltransferases such as methyltransferase-like 16 (METTL16), zinc finger CCCH-type containing 13 (ZC3H13), vir-like m6A methyltransferase associated (VIRMA) and RNA-binding motif protein 15/15B (RBM15/15B) have been reported to exhibit indispensable roles in the process of RNA m6A methylation in recent years [26–28].

In contrast to methyltransferases, m6A demethylases are vividly named as ‘m6A erasers’, and they act to remove methylation, thus making the m6A modification process dynamic and reversible. Until now, two FeII/α-KG-dependent dioxygenase AlkB family members, fat mass and obesity-associated protein (FTO) and alkB homolog 5 (ALKBH5), are identified as m6A erasers. In 2012, Jia et al. revealed that FTO was not only associated with human obesity [29], but also had oxidative demethylation activity of m6A residues in RNA [30]. In addition, ALKBH5 is the second identified m6A demethylase that can affect RNA nuclear export and metabolism. In male mice, the deficiency of ALKBH5 was found to increase m6A modification in mRNAs [31].

M6A modification can be recognized by specific m6A-binding proteins that serve as ‘m6A readers’. These proteins are able to bind to m6A sites or change RNA secondary structure to perform their biological functions [32]. The m6A readers mainly contain multiple YT521-B homology (YTH) domain family members (YTHDC1/2 and YTHDF1/2/3), heterogeneous nuclear ribonucleoproteins (HNRNPs) family proteins (HNRNPA2B1/C/G) and eukaryotic initiation factor 3 (eIF3), etc. [33]. They have a variety of functions, and participate in the regulation of RNA splicing, translation, transport, degradation, stability and processing. For example, YTHDC1 contributes to the RNA splicing and export [34, 35]. YTHDF2 recognizes m6A sites and drives RNA deregulation via two distinct pathways [36]. Recently, HNRNPA2B1 was reported to be inseparable from miRNA processing [37].

### Mutual regulation between m6A modification and circRNAs

CircRNAs are generally derived from precursor mRNAs (pre-mRNAs) and possess covalently closed single-stranded structures, which facilitate their resistance to RNase [38]. CircRNAs are classified into four categories, including exonic circRNAs (ecRNAs), exon-intron circRNAs (EIciRNAs), circular intronic RNAs (ciRNAs) and tRNA intronic circRNAs (tricRNAs), and their formation mechanisms are distinct. To date, several mechanisms have been reported for the biogenesis of circRNA, including lariat-driven circularization, RNA-binding proteins (RBPs)-associated circlarization, intron pairing-driven circularization and the bulge-helix-bulge (BHB) motif of precursor tRNAs-depended circularization [39–41]. Interestingly, circRNAs were initially regarded as useless by-products of pre-mRNAs. It was not until these years that people gradually uncovered that circRNAs are widely involved in the regulation of various aspects of many diseases, especially cancer [40, 42, 43]. The classic biological roles of circRNAs are that they can function as competing endogenous RNAs (ceRNAs) to sponge miRNAs and positively modulate downstream target genes expression. Specifically, circRNAs can competitively suppress miRNAs activity through miRNA response elements (MREs), leading to fewer miRNAs binding to 3′ untranslated regions (3′ UTRs) of target mRNAs, thereby relieving the inhibitory effects of miRNAs on mRNAs expression, and ciRS-7 was the first identified circRNA that act as a miRNA sponge [41, 44]. Furthermore, circRNAs also can act as regulators of their parental gene transcription, bind to proteins, and have unexpected translation potential [40].

**Fig. 2:**
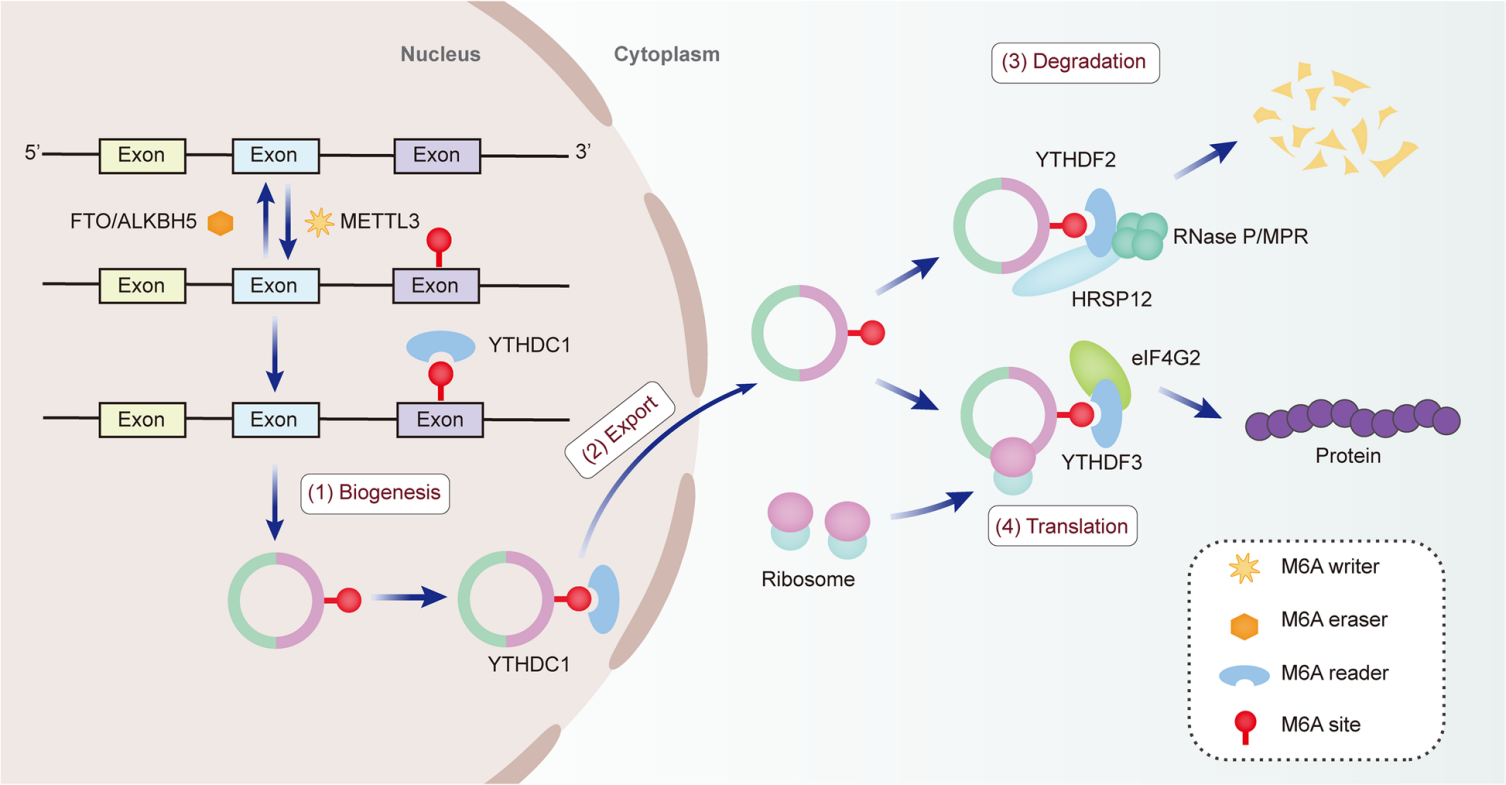
Impacts of m6A modification on biogenesis, export, degradation and translation of circRNAs. (1) Biogenesis: m6A sites located on pre-mRNAs are added by METTL3 (m6A writer), and can be identified by YTHDC1 (m6A reader), thus facilitating back-splicing and the biogenesis of circRNAs. (2) Export: the m6A reader YTHDC1 can also promote cytoplasmic export of circRNAs via interacting with m6A residues. (3) Degradation: m6A-modified circRNAs can be recognized by YTHDF2 (m6A reader), which participates in the formation of cleavage complex with HRSP12 and RNase P/MRP, resulting in the degradation of circRNAs. (4) Translation: YTHDF3 (m6A reader) and eIF4G2 are essential for the initiation of m6A-modified circRNAs translation

Strikingly, extensive studies have confirmed that m6A plays an indispensable role in the metabolism and functions of circRNAs [17]. Currently, the roles of m6A in circRNAs are mainly concentrated in four aspects (Fig. 2): (1) Biogenesis of circRNAs. Timoteo et al. revealed that m6A regulated circZNF609 biogenesis in an METTL3/YTHDC1-dependent manner [45]. Similarly, the formations of circ-ARL3, circMETTL3 and circ1662 have been demonstrated to be closely associated with m6A modification in multiple tumors [46–48]. However, the detailed mechanisms of m6A-dependent circRNAs biogenesis still need to be further explored. (2) Cytoplasmic export of circRNAs. Cytoplasmic export of circRNAs is essential for their functions. Some scholars have found that m6A-modified circNSUN2 in the nucleus could bind to YTHDC1, thus promoting its export to the cytoplasm [49]. Despite this breakthrough, more research about m6A-mediated cytoplasmic export of circRNAs is still required. (3) Degradation of circRNAs. CircRNAs are more stable than linear mRNAs due to their closed structure. Therefore, there are few reports on how circRNAs are degraded, and the specific mechanisms remain largely unclear. Recently, a new study revealed that circRNAs with m6A modification could be identified by YTHDF2, which participated in the formation of cleavage complexes with HRSP12 and RNaseP/MRP. Thus, the YTHDF2-HRSP12-RNase P/MRP axis exhibited a key role in circRNAs degradation [50, 51]. Additionally, m6A modification could inhibit the degradation of circRNAs, thus elevating circRNAs stability. For example, Wu et al. found that the half-life of m6A-modified circCUX1 was prolonged after treatment with Actinomycin D, indicating that m6A stabilized the expression of circCUX1 [52]. However, the molecular mechanisms by which m6A inhibits circRNAs degradation are unknown. (4) Translation of circRNAs. Most circRNAs are classified as ncRNAs, while a few circRNAs in the cytoplasm were found to possess peptides/protein-coding potential [53]. Because of the absence of 5′cap structure, the traditional cap-dependent translation model is not applicable to circRNAs. With continuous efforts, it is gradually known that circRNAs can be encoded via two cap-independent pathways, including the internal ribosome entry site (IRES) and m6A-driven translation [54, 55]. YTHDF3 and eIF4G2 are critical for the initiation of translating circRNAs containing m6A motifs. In addition, this m6A-mediated translation process can be inhibited by FTO and heightened by METTL3/14.

Conversely, aberrant expression of circRNAs also affect m6A modification. On one hand, circRNAs can indirectly modulate m6A regulators levels via sponging miRNAs. For instance, a recent data analysis suggested that circMAP2K4 interacted with miR-139-5p, which contributed to the target gene, YTHDF1, expression in hepatocellular carcinoma (HCC) [56]. Moreover, interacting with RBPs is also a significant function of circRNAs. Therefore, it is not surprise that some circRNAs, including circNOTCH1 and circPTPRA, can regulate the expression of target mRNAs through recruiting or competitively binding to m6A regulators [57–59]. Taken together, m6A modification not only regulates biogenesis and function of circRNAs, but is also affected by circRNAs.

## M6A modification involved in regulating cancer therapeutic resistance

**Fig. 3:**
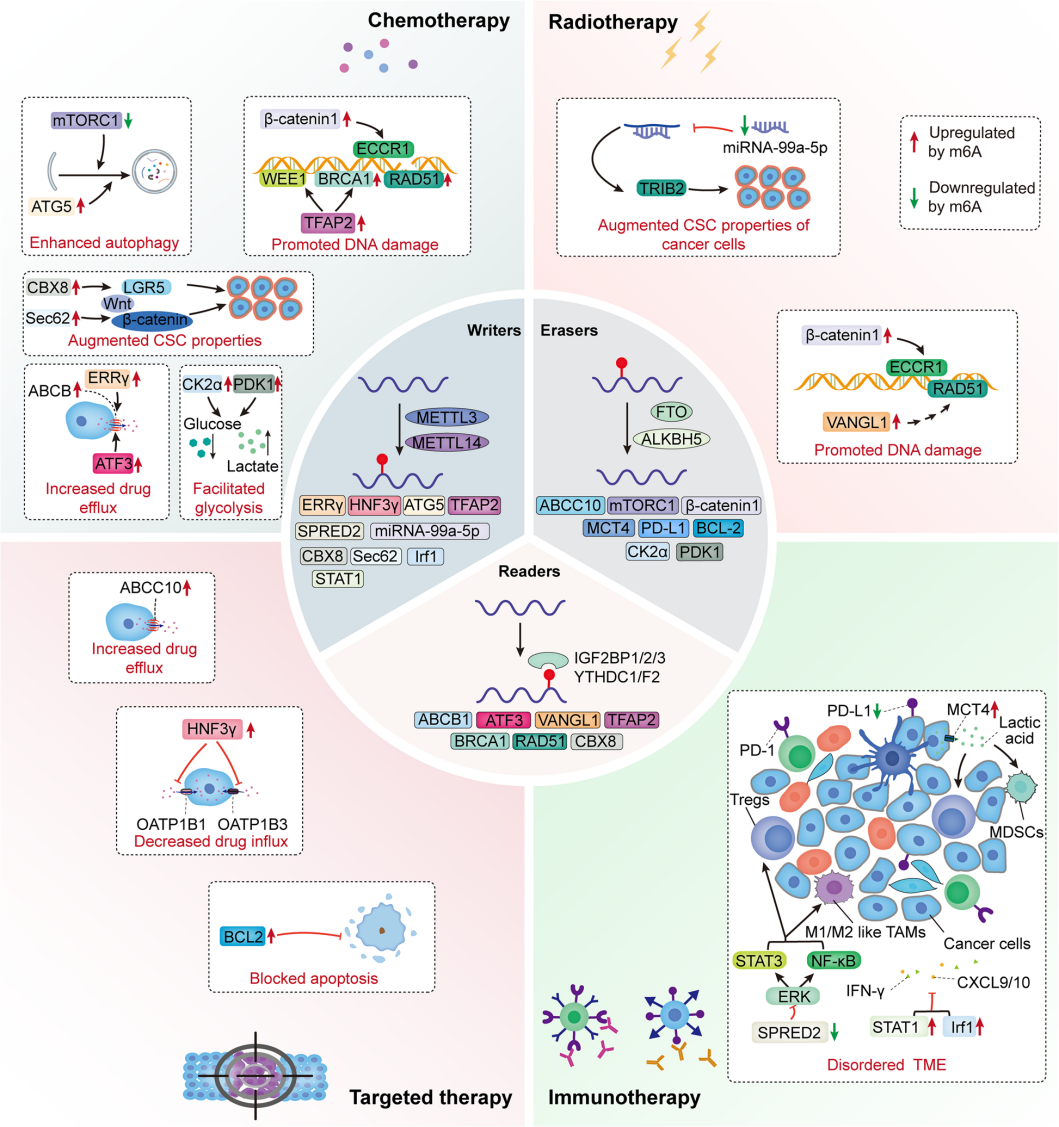
M6A modulators regulate therapeutic resistance in cancer. M6A modification is dynamically modulated by m6A writers, erasers and readers, and these m6A modulators play vital roles in therapeutic resistance. (1) In chemotherapy: m6A modification can reduce chemosensitivity through facilitating autophagy, DNA damage repair, CSC properties, drug efflux and glycolysis. (2) In radiotherapy: m6A modification can promote radioresistance via improving CSC properties and DNA damage repair. (3) In targeted therapy: m6A modification can increase drug efflux, suppress drug influx and apoptosis, thereby weakening the efficacy of target therapy. (4) In immunotherapy: m6A modification can remodel the TME, and further lead to immunotherapy resistance

In recent years, great breakthroughs and progress have been made in cancer treatment. However, many treatment strategies are not as effective as people might expect. The main reason for the unsatisfactory results is the emergence and development of therapeutic resistance in tumor cells. Various studies have shown that m6A modification is closely linked with cancer initiation, progression and therapeutic sensitivity, and m6A modulators play diverse roles in cancer therapy. Meanwhile, the molecular mechanisms are complicated and multifactorial. This section mainly summarizes that m6A modification regulates the response of cancers to therapy through different mechanisms (Table 1, Fig. 3).

**Table 1:** Altered m6A modification in cancer therapeutic resistance

Therapeutic resistance	Mechanisms	Cancer types	M6A regulators	Roles	Functions	Ref.
Chemoresistance	Drug transport	CRC	IGF2BP3↑	ABCB1↑	Doxorubicin resistance	[60]
		HCC/breast cancer	METTL3↑	ERRγ↑ → ABCB1↑	Doxorubicin resistance	[61]
		breast cancer	YTHDF2↓	ATF3↑ → ABCB1↑	Tamoxifen resistance	[62]
	Autophagy	GC	FTO↑	mTORC1 signal pathway↓	5-Fu/cisplatin/paclitaxel resistance	[63]
		Seminoma	METTL3↑	ATG5↑	Cisplatin resistance	[64]
	DNA damage repair	CSCC	FTO↑	β-catenin↑ → ECCR1↑	Cisplatin resistance and radioresistance	[13]
		breast cancer	YTHDC1↑	BRCA1/RAD51↑	Adriamycin resistance	[65]
		seminoma	METTL3/IGF2BP1↑	TFAP2C↑ → WEE1/BRCA1 ↑	Cisplatin resistance	[66]
	EMT and CSCs	CC	METTL3/IGF2BP1↑	CBX8↑ → LGR5↑	Oxaliplatin resistance	[67]
		CRC	METTL3↑	Sec62↑ → Wnt/β-catenin signaling↑	5-Fu/oxaliplatin resistance	[68]
	Glycolysis	BC	ALKBH5↓	CK2α↑	Cisplatin resistance	[69]
		GBM	FTO↑	PDK1↑	Temozolomide resistance	[70]
Radioresistance	DNA damage repair	LUAD	IGF2BP2/3↑	VANGL1↑ → BRAF/TP53BP1/RAD51 pathway↑	Radioresistance	[71]
	EMT and CSCs	ESCC	METTL14↓	miR-99a-5p↓ → TRIB2↑	Radioresistance	[72]
Targeted therapy resistance	Drug transport	NSCLC	FTO↑	ABCC10↑	Gefitinib resistance	[12]
		HCC	METTL14↓	HNF3γ↓ → OATP1B1/3↓	Sorafenib resistance	[73]
	Apoptosis	leukemia	FTO↑	BCL-2↑	TKIs resistance	[14]
Immunotherapy resistance	TME	–	METTL3↓	M1/M2-like TAM/Treg infiltration↑	Anti-PD-1 therapy resistance	[74]
		melanoma	ALKBH5↑	Tregs/MDSCs accumulation↑	Anti-PD-1 therapy resistance	[75]
		ICC	ALKBH5↓	PD-L1 ↓	Anti-PD-1/PD-L1 therapy resistance	[76]
		CRC and melanoma	METTL3/14↑	CD8+ T cells/CXCL9/CXCL10 ↓	Anti-PD-1 therapy resistance	[77]

### M6A-induced alterations in drug transport

For the efficacy of anti-cancer drugs, it is of great significance to study the intracellular drug concentrations. Drug transport mediated by drug transporters is a pivotal process that influences drug concentrations in tumor cells, and increased drug efflux and decreased drug influx are leading reasons for cancer drug resistance [5, 78].

Many anticancer drugs, such as cisplatin and paclitaxel, are substrates of ABC transporter family proteins that take charge of drug efflux [79, 80]. In general, ABC transporter family proteins consist of transmembrane domain (TMD) and adenosine triphosphate (ATP) binding region located in the cytoplasm [81]. After being combined with ATP, the ATP binding region transports the corresponding substrates to the membrane by hydrolyzing ATP. TMD is responsible for binding to the transported substrates, forming a transmembrane channel and helping them to pass through the membrane [82]. Recently, extensive reporters have demonstrated that m6A modification is a key player in the expression of ABC transporter family, that are strongly associated with multidrug resistance and thus indirectly regulate therapeutic resistance in cancer. Sulin-like growth factor 2 mRNA-binding protein 3 (IGF2BP3), as a new m6A reader, enhances target mRNA stability and nuclear export to regulate gene expression [83]. A current study suggested that overexpression of IGF2BP3 in HCT8/T cell bound to the m6A site of ABCB1 and elevated ABCB1 expression, thereby reducing the sensitivity of colorectal cancer (CRC) cells to chemotherapy [60]. In addition to direct regulation of m6A, the expression of ABCB1 was also regulated by m6A-modified estrogen receptor related receptors (ERRγ). In chemoresistant cancer cells, m6A modification-induced upregulation of ERRγ promoted chemoresistance through elevating ABCB1 level [61]. Moreover, Liu et al. surprisingly found that YTHDF2 regulated the expression of a transcription factor, ATF3, which further interacted with the enhancer of ABCB1 to stimulate its expression, leading to tamoxifen resistance [62]. ABCC10 serves as an efflux transporter of gefitinib, and affects the concentration of gefitinib in cells [84]. FTO was found to be enriched in serum exosomes from gefitinib-resistant patients, and overexpression of FTO upregulated ABCC10 in an m6A-dependent manner, and conferred non-small cell lung cancer (NSCLC) to gefitinib resistance [12].

Apart from ABC efflux transporter family, the expression of some organic anion transporting polypeptides (OATP) transporters belonging to the solute carrier superfamily responsible for drug uptake, is also affected by m6A modification. It is acknowledged that there are 11 members in OATP transporter family. Among them, OATP1B1 and OATP1B3 are specifically expressed on the basement membrane of human hepatocytes, and mediate drug transport in the liver [85]. Sorafenib, a targeted anticancer drug for the treatment of inoperable and distant metastatic HCC and advanced clear cell renal cell carcinoma (ccRCC), is recognized as a substrate of OATP1B1 and OATP1B3 [86, 87]. In a recent study, decreased hepatocyte nuclear factor 3γ (HNF3γ) level was measured in HCC tissues compared with adjacent normal tissues, and it was induced by reduction of METTL14, which further facilitated sorafenib resistance by inhibiting OATP1B1 and OATP1B3 expression [73].

### M6A modulates autophagy

Autophagy is a conserved intracellular degradation process that is a critical factor in adapting to metabolic stress and maintaining intracellular homeostasis [88]. Under certain stress conditions, cells can timely self-degrade damaged organelles and aggregated or dysfunctional proteins through autophagy mechanism, which is beneficial for the degradation of cells to gain survival advantage [89, 90]. Accordingly, autophagy induction in tumor cells may favor their response to anti-cancer treatment, thus reducing cell death and hastening therapeutic resistance.

Growing studies confirm that m6A modification has a hand in autophagy activation and the formation of autophagosomes responsible for loading some degradative substances [91]. For instance, FTO-mediated reduction of m6A modification on unc-51-like kinase 1(ULK1) suppressed YTHDF2-driven degradation of ULK1 transcripts, thereby promoting the initiation of autophagy [92]. In another study, FTO deficiency led to the degradation of ATG5 and ATG7, which acted as significant regulators in autophagosome formation, thus weakening autophagy [93]. In addition, Li et al. assessed the correlation of YTHDF1 with hypoxia-induced autophagy in vivo and in vitro. Subsequently, they found that overexpression of YTHDF1 could enhance autophagy under hypoxia by facilitating translation of two other autophagy-related genes, ATG2A and ATG14 [94]. Meanwhile, multiple tumor cells were demonstrated to be resistant to drug treatment, based on the mechanisms of autophagy induction under hypoxia [95–97]. Intriguingly, it was also revealed that omeprazole could augment m6A level in gastric cancer (GC) cells through eliminating FTO, and further studies found that reducing FTO attenuated pro-survival autophagy levels, thereby sensitizing GC cells to chemotherapy via boosting the activation of mTORC1 signaling pathway [63]. Hence, FTO is expected to be a potential therapeutic target to improve drug sensitivity in GC patients. METTL3 and METTL14, as important functional components of MTC, also play vital roles in the modulation of autophagy. A recent evidence shed light on the mechanism by which METTL3 reduced the sensitivity of seminoma to cisplatin by heightening autophagy [64].

### Role of m6A modification in DNA damage repair

Genomic instability caused by multiple factors, such as DNA damage, often leads to oncogenesis. Meanwhile, it also provides opportunities for cancer treatment [98]. The important ways of cancer therapy including chemotherapy and radiotherapy induce DNA damage so as to cause cell death. Nevertheless, tumor cells can activate their own DNA damage repair mechanisms, and ultimately become resistant to DNA-damaging drugs and radiation, resulting in therapy failure [99, 100]. DNA damage repair methods chiefly consist of nucleotide excision repair (NER), base excision repair (BER), homologous recombination repair (HRR) and non-homologous end joining (NHEJ), etc. [98].

It has been proved that some DNA damage repair proteins are essential players in chemoradiotherapy resistance. Moreover, the expression of these proteins is closely related to m6A modification. For example, excision repair cross-complementation group-1 (ERCC1), located on human chromosome 19, is a key gene in the NER pathway [101]. Its expression is positively correlated with cisplatin resistance in various cancers, including GC and cervical cancer [102, 103]. To broaden the horizons of FTO function in chemoradiotherapy resistance, Zhou et al. measured the expression of FTO and uncovered its regulatory role in cisplatin resistance and radioresistance. The results showed that FTO elevated the expression of β-catenin, which in turn caused the upregulation of ECCR1. Subsequently, increased ECCR1 gave rise to failure of cisplatin therapy and radiotherapy in cervical squamous cell carcinoma (CSCC) via activating NER [13]. DNA double strand breaks (DSBs) are the most deadly DNA damage, however, it can be repaired by HRR, which is a slow but highly accurate repair process [104]. Aberrant expression of key genes therein may result in altered sensitivity of tumor cells to anti-cancer therapy. One suitable example is that increased YTHDC1 mediated by epithelial membrane protein 3 (EMP3) downregulation exhibited a positive effect on the expression of BRCA1 and RAD51, thereby promoting DNA repair in breast cancer cells, leading to chemoresistance [65]. Additionally, VANGL1 was upregulated upon increased m6A levels in VANGL1 mRNA and miR-29b-3p depletion. The upregulated VANGL then inhibited the harmful influence of radiation on lung adenocarcinoma (LUAD) by activating BRAF/TP53BP1/RAD51 pathway correlated with DNA repair [71]. Interestingly, enhanced stability of TFAP2C induced by IGF2BP1 contributed to the activation of WEE1 and BRCA1 in cisplatin-resistant seminoma cells [66].

### Remodeling of TME by m6A regulators

The TME is an indispensable soil for tumor growth and development. It is a complex and multicomponent system that contains several types of cells, such as cancer-associated fibroblasts and immune cells, as well as other biochemical factors such as cytokines and chemokines, etc. [105]. In recent years, emerging immunotherapy achieves the purpose of anti-tumor by stimulating or rebuilding immune system, which has brought tremendous progress in the treatment of malignant tumors [106]. With the increasing recognition of the complexity and variability of TME, people gradually realize the important role of TME in tumor immunotherapy. For example, immunosuppression in the TME is caused by multiple factors such as elevated immune checkpoints, recruitment of more immunosuppressive cells such as T regulatory cells (Tregs), myeloid-derived suppressor cells (MDSCs) and tumor-associated macrophages (TAMs), and increased production of immunosuppressive molecules limits the efficacy of immunotherapy, leading to therapeutic resistance [107–110]. Tregs infiltration directly inhibits cytotoxic T cells proliferation and produces immunosuppressive cytokines such as IL-10, IL-35 and transforming growth factor β (TGF-β), thereby supporting tumor immune escape and potentiating tumor progression [111]. Another study exhibited that increased MDSCs and chemokines within the TME could promote the development of resistance to dual ICIs in BRAF inhibitor-resistant melanoma [112]. Not surprisingly, m6A modification can affect the sensitivity of various tumor cells to immunotherapy via taking part in the regulation of anti-cancer immune responses within the TME. Yin et al. revealed that METTL3 deficiency facilitated M1/M2-like TAM and Treg infiltration to attenuate the response to anti-PD-1 therapy [74]. Similarly, ALKBH5 knockout had a positive influence on anti-PD-1 treatment, relying on the inhibition of normal lactate transport and accumulation of Tregs and MDSCs [75]. Moreover, ALKBH5-mediated reduction of m6A modification of PD-L1 suppressed PD-L1 degradation, and its upregulation could improve the sensitivity of intrahepatic cholangiocarcinoma (ICC) cells to anti-PD-1/PD-L1 immunotherapy [76]. Recently, it was found that METTL3/14 knockdown elevated the efficacy of immunotherapy. Mechanistically, CD8+ T cells and chemokines including CXCL9 and CXCL10 were significantly upregulated upon METTL3/14 deletion, depending on enhanced IFN-c-Stat1-Irf1 signaling pathway in CRC and melanoma [77].

### The influence of m6A on EMT and cancer stem cells

In 1982, the concept of EMT was firstly proposed by Greenberg et al. when they found that epithelial cells cultured in three-dimensional collagen gels lost their polarity and exhibited a mesenchymal phenotypes [113]. With further recognition of EMT, it has been observed that EMT not only occurs throughout embryonic development and wound healing, but also plays a key role in various aspects of tumor progression through enhancing tumor cells migration, invasion and anti-apoptosis abilities [114]. To date, plenty of studies have suggested that EMT is involved in therapeutic resistance of various tumors, including breast cancer, lung cancer and ovarian cancer [115–117]. As a dynamic process, EMT-enhanced cells exhibit changes in molecular expression levels, such as reduction in epithelial markers (e.g. E-cadherin) and increase in EMT transcription factors (e.g. Snail, Slug and Twist). During EMT, m6A modification is also involved in the modulating these changes. A representative example is that overexpression of METTL14 prominently stimulated Twist expression and suppressed E-cadherin expression, which would lead to the enhancement of EMT [118]. Besides, Yu et al. analyzed the expression profile of m6A-related regulators acquired from TCGA database, and revealed that upregulated IGF2BP2 in head and neck squamous cell carcinoma (HNSCC) helped stabilize Slug mRNA, thus facilitating MET process and lymphatic metastasis of HNSCC [119]. Consistently, EMT regulated by m6A has also been observed in other cancers, including GC and breast cancer [120, 121]. Taken together, these studies imply that m6A modification may contribute to therapeutic resistance by influencing EMT process.

Strikingly, the activation of EMT is closely associated with the generation of cancer stem cells (CSCs) [122]. CSCs resemble embryonic stem cells with unlimited self-renewal and differentiation potential. These properties can confer tumor cells the ability to tolerate detrimental stress, such as damage caused by chemotherapy drugs and radiation [123]. M6A-induced upregulation of CBX8 could enhance stemness and chemoresistance of colon cancer (CC) through combing with KMT2b and Pol II to promote LGR5 transcription [67]. Moreover, m6A modification could facilitate the acquisition of CSCs properties via promoting Wnt/β-catenin signaling, thereby inhibiting chemosensitivity of CRC [68]. Similarly, CSCs generation and radiosensitivity in esophageal squamous cell carcinoma (ESCC) were also influenced by m6A methyltransferase METTL14 [72].

### Other mechanisms mediated by m6A regulators

Indeed, there are several other mechanisms related to cancer therapeutic resistance, including alterations in drug-metabolizing enzymes, abnormal apoptosis, glycolysis, and so on. However, the role of m6A modification in these mechanisms is not fully understood and still under continuous investigation. Some chemotherapeutic prodrugs, such as cyclophosphamide and 5-fluorouracil (5-FU), need to be converted into active drugs by drug-metabolizing enzymes before binding to their targets, and drug-metabolism can also inactivate these anti-cancer drugs, indicating that drug-metabolizing enzymes are critical to drug efficacy [124]. An attractive report showed that mRNA levels of cytochrome (CYP) P450 family members (CYP1A2, CYP2B6, and CYP2C8) were remarkably elevated following treatment with RNA methylation inhibitor. Follow-up experiments demonstrated that METTL3/14 overexpression or FTO deficiency could facilitate CYP2C8 mRNA degradation, implying that m6A regulators may affect drug efficacy in cancer therapy by regulating drug-metabolizing enzymes [125].

Apoptosis, a controlled cell death process, participates both in regulating normal physiological processes and in the progression of some diseases [126]. Evasion of apoptosis often occurs in tumor cells and has become one of the important hallmarks of treatment failure. The proteins involved in apoptosis mainly include anti-apoptotic B-cell lymphoma-2 (BCL-2) family members (e.g. BCL-2, MCL-1 and BCL-XL), pro-apoptotic BCL-2 proteins (e.g. BAX and BAK) and inhibitors of apoptosis proteins (IAPs), etc. [127]. Interestingly, m6A modification was found to modulate anti-apoptotic BCL-2 family proteins expression. For example, the m6A writer METTL3 enhanced BCL-2 expression in breast cancer, leading to inhibition of apoptosis [128]. Notably, BCL-2 mRNA level was strongly associated with multiple drug resistance including paclitaxel, tamoxifen and trastuzumab [129–131]. Likewise, overexpression of FTO could promote BCL-2 expression, thereby reducing the sensitivity of leukemia cells to tyrosine kinase inhibitors (TKIs) [14].

Altered energy metabolism is now known as one of the remarkable hallmarks of cancer. In 1924, Otto Warburg firstly uncovered that tumor cells preferentially relied on glycolysis for energy production, even under aerobic conditions [132]. This type of energy metabolism is called ‘aerobic glycolysis’ or ‘Warburg effect’. During aerobic glycolysis, tumor cells swiftly consume glucose to produce lactic acid and ATP. Although glycolysis produce much less ATP than oxidative phosphorylation, this quick energy production is more advantageous for rapid proliferation of tumor cells [133]. Additionally, plenty of lactic acid promote the formation of acidic TME, and glycolytic metabolic intermediates can be used as precursors for the synthesis of certain biomacromolecules, both of which contribute to tumor growth, distant metastasis and therapeutic resistance [134]. Recently, Yu’s group discovered that ALKBH5 depletion increased casein kinase 2 α (CK2α) expression, indicating that m6A modification promoted cisplatin resistance in bladder cancer (BC) by upregulating CK2α-mediated glycolysis pathway [69]. Similarly, lncRNA JPX facilitated aerobic glycolysis through the FTO/PDK1 axis, thus conferring resistance to temozolomide in glioblastoma multiforme (GBM) cells [70].

## CircRNAs and cancer therapeutic resistance

**Fig. 4:**
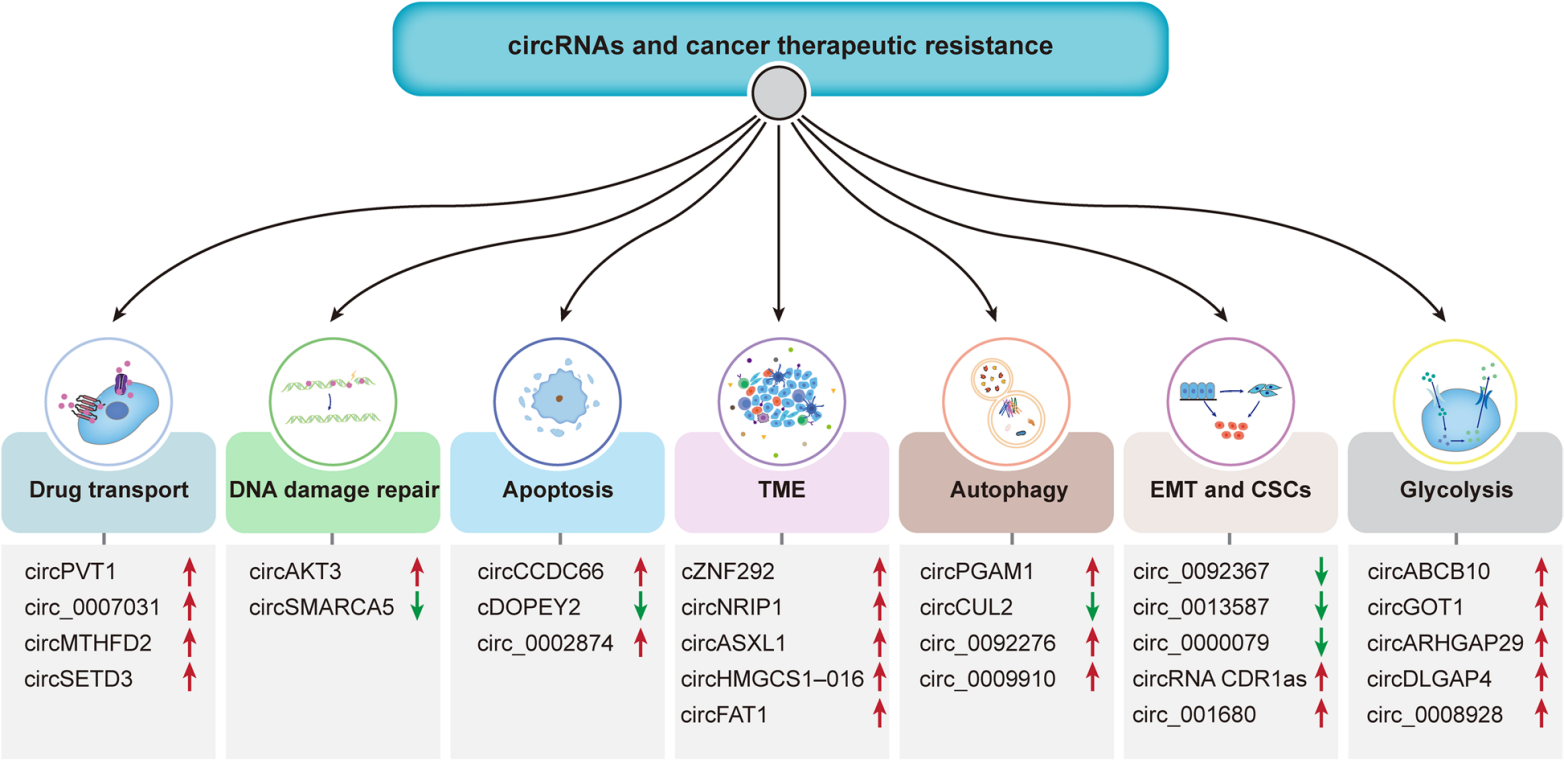
CircRNAs associated with cancer therapeutic resistance. CircRNAs can affect sensitivity to cancer therapy through multiple mechanisms, including modulation of drug transport (such as circPVT1 and circ_0007031), DNA damage repair (such as circAKT3 and circSMARCA5), apoptosis (such as circCCDC66 and cDOPEY2), TME (such as cZNF292 and circNRIP1), autophagy (such as circPGAM1 and circCUL2), EMT and CSCs (such as circ_0092367 and circ_0013587) and glycolysis (such as circABCB10 and circGOT1)

Accumulating understanding about circRNAs has elucidated the vial roles of circRNAs in the proliferation, migration and invasion of various tumor cells, including breast, cervical and HCC cancer cells [135–137]. In recent decades, attention has increasingly focused on therapeutic resistance. Growing evidence suggests that circRNAs affect cancer treatment sensitivity through multiple mechanisms, including modulating drug transport, DNA repair, apoptosis, TME, autophagy, EMT, CSCs and glycolysis (Table 2, Fig. 4).

**Table 2:** CircRNAs involved in cancer therapeutic resistance

Therapeutic resistance	Mechanisms	Cancer types	CircRNAs	Roles	Functions	Ref.
Chemoresistance	Drug transport	LUAD	circPVT1↑	miR-145-5p↓ → ABCC1↑	Cisplatin/pemetrexed resistance	[138]
		CRC	circ_0007031↑	miR-133b↓ → ABCC5↑	5-FU resistance	[139]
		GC	circMTHFD2↑	miR-124↓ → ABCB1↑	Pemetrexed resistance	[140]
	DNA damage repair	GC	circAKT3↑	PI3K/AKT pathway↑ → BRCA1↑	Cisplatin resistance	[141]
		breast cancer	circSMARCA5↓	SMARCA5↑	Cisplatin resistance	[142]
	Apoptosis	GC	circCCDC66↑	miR-618↓ → BCL-2↑	Cisplatin resistance	[143]
		EC	cDOPEY2↓	CPEB4↑ → MLC-1↑	Cisplatin resistance	[144]
		NSCLC	circ_0002874↑	miR-1273f↓ → MDM2↑ → P53↓	Paclitaxel resistance	[145]
	TME	GC	circNRIP1↑	miR-138-5p↓ → HIF-1α↑	5-FU resistance	[146]
		NSCLC	circASXL1↑	miR-206↓ → HIF-1α↑	Cisplatin resistance	[147]
	Autophagy	laryngocarcinoma	circPGAM1↑	miR-376a↓ → ATG2A↑	Cisplatin resistance	[148]
		GC	circCUL2↓	miR-138-5p↑ → ROCK2↓	Cisplatin resistance	[149]
		breast cancer	circ_0092276↑	miR-384↓ → ATG7↑	Doxorubicin resistance	[150]
	EMT and CSCs	PC	circ_0092367↓	miR-1206↑ → ESRP1↓	Gemcitabine resistance	[151]
		NSCLC	circ_0000079↓	FXR1/PRCKI complex↑	Cisplatin resistance	[152]
		NSCLC	circRNA CDR1as↑	HOXA9↓ → miR-641↑	Cisplatin resistance	[153]
		CRC	circ_001680↑	miR-340↓ → BMI1↑	Irinotecan resistance	[154]
	Glycolysis	ESCC	circGOT1↑	miR-606↓ → GOT1↑	Cisplatin resistance	[155]
		prostate cancer	circARHGAP29↑	c-Myc↑ → LDHA↑	Docetaxel resistance	[156]
		neuroblastoma	circDLGAP4↑	miR-143↓ → HK2↑	Doxorubicin resistance	[157]
		NSCLC	circ_0008928↑	miR-488↓ → HK2↑	Cisplatin resistance	[158]
Radioresistance	TME	HCC	cZNF292↑	SOX9 nuclear translocation↑ → Wnt/β-catenin pathway↑	Radioresistance	[159]
	Glycolysis	breast cancer	circABCB10↑	miR-223-3p↓ → PFN↑	Radioresistance	[160]
Targeted therapy resistance	Drug transport	NSCLC	circSETD3↑	miR-520 h↓ → ABCG2↑	Gefitinib resistance	[161]
	Autophagy	CML	circ_0009910↑	miR-34a-5p↓ → ULK1↑	Imatinib resistance	[162]
	EMT and CSCs	PC	circ_0013587↓	miR-1227↑ → E-cadherin↓	Erlotinib resistance	[163]
Immunotherapy resistance	TME	ICC	circHMGCS1–016↑	miR-1236-3↓ → CD73/GAL-8↑	Anti-PD-1 therapy resistance	[164]
		HNSCC	circFAT1↑	STAT3↑ → CD8+ T cells infiltration↓	Anti-PD-1 therapy resistance	[165]

### CircRNAs and drug transport

Accumulating studies have suggested that certain circRNAs can affect the levels of ABC transporters in drug-resistant tumor cells. For example, Zheng’s group revealed that circPVT1 could promote ABCC1 expression via binding to miR-145-5p, resulting in a reduced accumulation of cisplatin and pemetrexed in LUAD cells [138]. Consistently, ABCB1 expression in osteosarcoma cells was also modulated by circPVT1 [166]. One study involved circ_0007031, which contributed to 5-FU resistance of CRC cells through miR-133b/ABCC5 pathway [139]. In pemetrexed-resistant GC, overexpression of circMTHFD2 can sponge miR-124 and increase the expression of ABCB1 [140]. Additionally, the circSETD3/miR-520 h/ABCG2 axis was disclosed to be strongly associated with the sensitivity of NSCLC cells to gefitinib [161]. Unfortunately, studies on the roles of circRNAs in regulating drug uptake have not been reported.

### CircRNAs and DNA damage repair

DNA damage repair is an important mechanism of chemoresistance and radioresistance. Wang et al. uncovered that low-dose radiation was able to induce the secretion of exosomes rich in circ-METRN. The increased circ-METRN upregulated DNA damage molecule γ-H2AX expression, but γ-H2AX level returned to normal at 24 h, indicating enhanced DNA damage repair process in radioresistant glioblastoma cells [167]. Moreover, in cisplatin-resistant GC, the PI3K/AKT pathway, which positively regulates BRCA1 expression, can be activated by circAKT3 [141, 168]. Recently, circRNAs were found to regulate corresponding parent genes expression by binding to their cognate DNA locus to form R-loops [169]. This novel function of circRNAs has also been demonstrated in cancer. Namely, the interaction of circSMARCA5 with SMARCA5 could suppress the expression of SMARCA5, which is vital for DNA damage repair, and altered DNA damage repair modulated the sensitivity of breast cancer cells to cytotoxic drugs [142, 170]. In summary, these findings disclose the importance of circRNAs in cancer therapy by regulating DNA damage repair.

### CircRNAs and apoptosis

For cancer, most therapy strategies can efficiently eliminate part of tumor cells by inducing apoptosis. However, dysregulation of apoptotic signaling is instrumental to tumor cell survival and leads to therapeutic resistance. In recent years, evidence is accumulating to show that many circRNAs modulate apoptosis process in tumor cells via participating in the regulation of apoptosis-related proteins and signaling pathways. For examples, circCCDC66 was detected to be upregulated in cisplatin-resistant GC cells and tissues. CircCCDC66 depletion elevated cisplatin sensitivity due to its interaction with miR-618 to inhibit BCL-2 expression [143]. Liu’s group disclosed that loss of circDOPEY2 promoted translation of anti-apoptosis protein MLC-1 by decreasing ubiquitination and degradation of CPEB4. This function ultimately suppressed apoptosis and triggered chemoresistance of esophageal cancer (EC) cells [144]. BIRC3, also known as cellular IAP-2, belongs to the IAPs family and exerts a pro-survival and anti-apoptotic roles in tumors [171]. A recent study delineated that circDOCK1 could increase the expression of BIRC3 by sequestering miR-196a-5p, implying that circDOCK1 may be a significant biomarker and a valuable therapeutic target for oral squamous cell carcinoma in the future [172]. Besides, hsa_circ_0110757 could function by activating PIK/Akt pathway through hsa-miR-1298-5p/ITGA axis in glioma [173]. Noteworthily, previous reports have corroborated the close relationship between PIK/Akt signal pathway and BCL-2 expression, indicating that hsa_circ_0110757 might augment glioma resistance to temozolomide through inhibiting apoptosis [174, 175]. The tumor suppressor gene p53 is significant for apoptosis induction. Xu et al. uncovered that upregulated hsa_circ_0002874 could inhibit p53 expression via miR-1273f/MDM2 axis, leading to apoptosis inhibition and paclitaxel resistance in NSCLC [145].

### CircRNAs and TME

Growing facts have elucidated that a plethora of circRNAs exhibit critical roles in multiple aspects of TME, such as angiogenesis, hypoxia, endothelial monolayer permeability (ECM) and immune surveillance [176]. Hypoxia is a typical feature of TME. The rapid proliferation of tumor cells promotes massive oxygen consumption and abnormal proliferation of tumor vascular structure, causing an imbalance between tumor oxygen supply and oxygen demand [177]. Hypoxic TME can confer greater viability of tumor cells during radiation and drug therapy by affecting drug efflux proteins expression, mitochondrial activity, apoptosis, autophagy and EMT [178]. It was reported that the expression of cZNF292 was strongly associated with hypoxia-induced radioresistance. Data from mechanism experiments suggested that cZNF292 deficiency elevated SOX9 nuclear translocation, which inhibited the activation of Wnt/β-catenin pathway, thereby enhancing the radiosensitivity of hypoxic HCC cells [159]. Consistently, some circRNAs, such as circNRIP1 and circASXL1, have been demonstrated to modulate hypoxia-associated resistance in chemotherapy [146, 147]. In addition, circRNAs can influence the efficiency of immunotherapy by regulating anti-tumor immunity [164, 165, 179]. For instance, Xu et al. disclosed that upregulation of circHMGCS1–016 promoted immunosuppression via sponging miR-1236-3 to increase CD73 and GAL-8 expression, which further contributed to the inhibition of CD8+ T and CD4+ T cells, thereby facilitating anti-PD-1 resistance in ICC [164]. Another example is that circFAT1 deletion improved the efficacy of anti-PD-1 immunotherapy through impairing cancer stemness and elevating CD8+ T cells infiltration into the TME. This effect was based on the role of circFAT1 in STAT3 activation [165].

### CircRNAs and autophagy

Autophagy is an auto-catabolic process that can assist tumor cells to deal with therapeutic pressure and escape crisis [180]. To better address acquired resistance, having a clear understanding of the regulatory relationship between circRNAs and autophagy is of great importance. The regulatory roles of circRNAs in autophagy are mainly based on their function as miRNA sponges. CircPGAM1 was instrumental to drug resistance in laryngocarcinoma, whereas overexpression of miR-376a reversed this effect via decreasing ATG2A level [148]. CircCUL2 deficiency enhanced activation of autophagy by miR-142-3p/ROCK2 axis, thus promoting GC progression and cisplatin resistance [149]. Gao et al. have delineated that hsa_circ_0092276 was upregulated in doxorubicin-resistant breast cancer cells [181]. Another research team further uncovered that hsa_circ_0092276 increased ATG7 expression through binding to miR-384, leading to enhanced autophagy and doxorubicin resistance [150]. Moreover, the ULK complex made up of ULK1 or ULK2, mATG13 and FIP200 is important for autophagy activation [182]. A recent study found that ULK1 was highly expressed in imatinib-resistant chronic myeloid leukemia (CML) cells. The upregulation of ULK1 was mediated by the action of circ_0009910 in sponging miR-34a-5p [162]. Notably, further studies of circRNAs regulating autophagy-related therapeutic resistance in cancer through other biological functions are needed.

### CircRNAs and EMT and CSCs

In recent years, increasing studies have suggested that abnormal expression of circRNAs can enhance EMT and endow tumor cells with therapeutic resistance. A typical instance is circ_0092367, whose depletion dampened epithelial splicing regulatory protein 1 (ESRP1) expression by acting as miR-1206 sponger. The downregulated ESRP1 promoted EMT and further contributed to chemoresistance to gemcitabine in PC [151]. Erlotinib has also been demonstrated to possess potential to treat PC patients. However, the development of acquired drug resistance would restrict its therapeutic efficacy. New evidence uncovered by Xu et al. show that the upregulation of circ_0013587 reversed erlotinib resistance in vitro and in vivo by binding to miR-1227 to increase E-cadherin expression [163]. Circ_0000079, derived from human USP1 gene, was markedly reduced in NSCLC patients resistant to cisplatin, which repressed EMT-induced chemoresistance by decoying FXR1 to block the formation of the FXR1/PRCKI complex in NSCLC [152].

Importantly, the resistance mechanisms associated with CSCs are also regulated by certain circRNAs. For instance, Zhao et al. found enhanced circRNA CDR1as and stemness markers, including SOX2, Nanog and OCT4, in cisplatin-resistant NSCLC cells. Mechanistically, CDR1as overexpression elevated stemness of cisplatin-resistant NSCLC cells through regulating miR-641/HOXA9 axis [153]. Moreover, circ_001680 upregulated the mRNA and protein level of BMI1 by sequestering miR-340, which further promoted the CSC population in CRC, thus accelerating cell proliferation, migration and irinotecan resistance [154]. Overall, clarification of the tight relationship between circRNAs and CSCs is vital in overcoming cancer therapeutic resistance.

### CircRNAs and glycolysis

The energy metabolism of tumor cells is significantly different from that of normal cells. For tumor cells, they prefer to generate ATP and lactic acid through the glycolytic pathway, which has been proved to induce cancer chemoradiation resistance. Extensive evidence has shown that some circRNAs are involved in glycolysis-induced chemoradiotherapy resistance in cancer. For instance, upregulation of circABCB10 augmented glycolytic metabolism to promote radioresistance by miR-223-3p/PFN axis in breast cancer. Breast cancer cells treated with glycolysis inhibitor 2-deoxy-D-glucose were found to be more sensitive to radiation than controls [160]. Notably, circGOT1 and its host gene GOT1 were identified to be upregulated in ESCC cells and tissues. Further mechanistic experiments showed that circGOT1 interacted with miR-606 to upregulate GOT1 expression, thereby facilitating aerobic glycolysis and cisplatin resistance [155]. Besides, lots of glycolysis-related genes expression were modulated by circRNAs, such as glucose transporter-1, lactic dehydrogenase A (LDHA) and hexokinases (HKs) [156–158, 183]. Some time ago, Jiang et al. found that circARHGAP29 upregulated LDHA level via binding to and stabilizing c-Myc, resulting in increased glycolysis-mediated docetaxel resistance in prostate cancer cells [156]. HKs are involved in catalyzing the first step of glucose metabolism, and play essential roles in aerobic glycolysis in tumor cells. It was reported that exosome-delivered circDLGAP4 elevated HK2 expression by sponging miR-143, and the increased HK2 further enhanced glycolysis in doxorubicin-resistant neuroblastoma cells [157]. Consistently, the role of circ_0008928 in cisplatin resistance was positively modulating HK2 via miR-488 [158].

## The interplay between m6A modification and circRNAs in cancer therapeutic resistance

### M6A-modified circRNAs in cancer therapeutic resistance

**Fig. 5:**
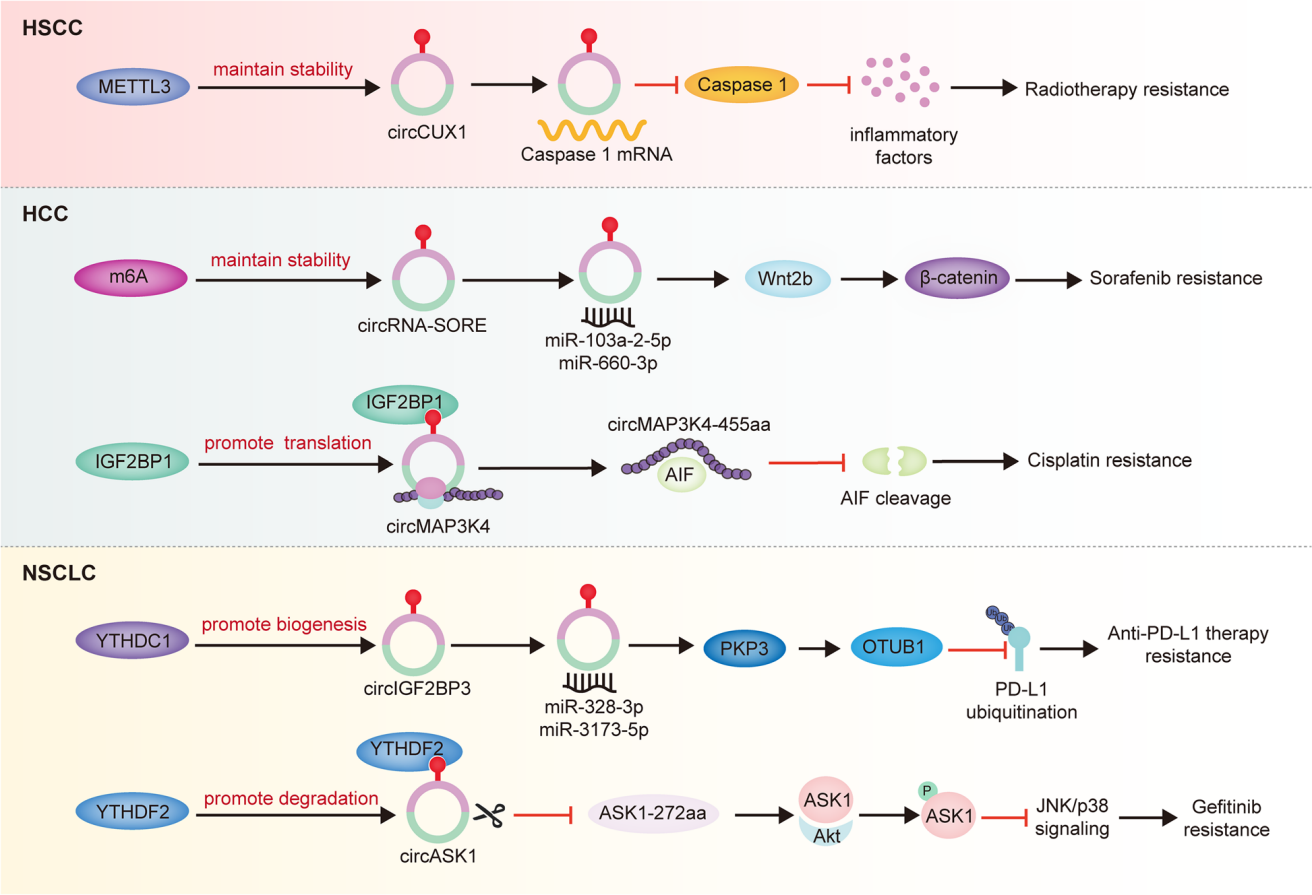
The role of m6A-modified circRNAs in cancer therapeutic resistance. M6A modification affects the biogenesis, translation and degradation of circRNAs, thus regulating the roles of circRNAs in therapeutic resistance of multiple cancers. (1) In HSCC: METTL3 elevated circCUX1 stability, leading to radioresistance. (2) In HCC: m6A regulators promoted circRNA-SORE stability and circMAP3K4 translation, resulting in sorafenib and cisplatin resistance, respectively. (3) In NSCLC: YTHDC1 and YTHDF2 facilitated anti-PD-L1 therapy and gefitinib resistance via increasing circIGF2BP3 biogenesis and circASK1 degradation, respectively

With the increasing understanding of circRNAs and m6A modification, it has been gradually uncovered that m6A is widely present in circRNAs, and involved in modulating their biogenesis, localization, degradation and translation. Since both circRNAs and m6A are key modulators in cancer therapeutic resistance, m6A modification is likely to affect the biological functions of circRNAs in cancer treatment resistance. Herein, we briefly summarized the recent research on the regulatory roles of m6A-modificated circRNAs in the treatment tolerance of malignancies such as HCC, hypopharyngeal squamous cell carcinoma (HSCC) and NSCLC (Table 3, Fig. 5).

**Table 3:** M6A-modified circRNAs in cancer therapeutic resistance

Cancer types	M6A regulators	Roles of m6A in circRNAs	Functions	Mechanisms	Ref.
HSCC	METTL3	Promotes m6A modification of circCUX1 to stabilizes its expression	Radiotherapy resistance	Decreases the release of inflammatory factors in TME	[52]
HCC	m6A	Elevates circRNA-SORE expression via increasing RNA stability	Sorafenib resistance	Activates the Wnt/β-catenin pathway	[184]
	IGF2BP1	Promotes m6A-modified circMAP3K4 translation	Cisplatin resistance	Inhibits apoptosis	[185]
NSCLC	YTHDC1	Facilitates the biogenesis of m6A-modified circIGF2BP3	Anti-PD-L1 therapy resistance	Promotes tumor immune evasion	[186]
	YTHDF2	Increases m6A-modified circASK1 degradation	Gefitinib resistance	Represses apoptosis	[51]

#### Hypopharyngeal squamous cell carcinoma (HSCC)

HSCC is a common malignant tumor originating from the mucous epithelium of the upper aerodigestive tract, accounting for about 5–15% of head and neck squamous cell carcinoma, and is characterized by hidden location, atypical early symptoms, high early metastasis rate and easy recurrence [187, 188]. Consequently, identifying the mechanisms that upregulate progression and therapy tolerance at the molecular level will aid to improve the diagnosis and treatment of HSCC. Li et al. discovered upregulated circ_0058106 in HSCC tissues, and further elucidated that increased circ_0058106 positively modulated Wnt2b/β-catenin/c-Myc pathway by sponging miR-185-3p, thus giving rise to enhanced tumorigenesis and EMT in HSCC [189]. Consistently, upregulated circMATR3 could potentiate the proliferation, migration, invasion and repress apoptosis in HSCC cells [188]. However, what promotes the expression of circ_0058106 and circMATR3 remains unclear. It is likely that m6A modification has a regulatory effect on circRNA expression level in HSCC. Recently, Wu et al. provided strong evidence to support this hypothesis. They were surprised to find that METTL3-mediated m6A methylation of circCUX1 stabilized its expression. CircCUX1 further suppressed caspase 1 expression via interacting with it, which dampened the release of inflammatory factors (IL-1β and IL-18) in TME, thus contributing to radiotherapy tolerance of HSCC [52].

#### Hepatocellular carcinoma (HCC)

As the main subtype of primary liver cancer, HCC comprises 75–85% of cases, which is one of the leading causes of cancer-related deaths worldwide [46]. In recent years, accumulating evidence has confirmed that circRNAs and m6A, especially their interplays, play key roles in many aspects of HCC [190]. For instance, METTL3 and YTHDC1 were uncovered to promote circHPS5 generation and cytoplasmic export in HCC, respectively. Functionally, m6A-modulated circHPS5 enabled to upregulate HMGA2 expression through sponging miR-370, thereby improving EMT and CSC phenotypes to potentiate HCC development [191]. Sorafenib is an FDA-approved target drug for advanced HCC, but acquired resistance has impedes its clinical application. CircRNA-SORE transmitted by exosomes was upregulated in sorafenib-resistant HCC cells, and dampened PRP19-induced YBX1 degradation through binding to YBX1, which decreased the therapeutic efficacy of sorafenib [192]. Another report revealed that m6A could favor stabilizing the expression of circRNA-SORE, which potentiated the activation of Wnt/β-catenin pathway by serving as miR-103a-2-5p and miR-660-3p sponges [193]. Additionally, Wnt/β-catenin pathway helped tumor cells to acquire stem cell properties with unlimited self-renewal and differentiation potential [184], contributing to sorafenib tolerance. More interestingly, m6A modification also participates in the translation of circRNAs in HCC. Duan et al. uncovered that circMAP3K4 could be encoded into circMAP3K4-455aa in an m6A-mediated manner, which protected AIF from cleavage, leading to decreased cisplatin-induced apoptosis in HCC cells [194].

#### Non-small-cell lung cancer (NSCLC)

Lung cancer poses a serious threat to human health and contributes the most to cancer-related deaths worldwide. NSCLC is histologically broadly classified into three types: LUAD, squamous adenocarcinoma and large-cell carcinoma, and is the most common subtype of lung cancer [185]. Since the efficacy of traditional radiotherapy and chemotherapy are compromised, most patients with intermediate and advanced NSCLC have high mortality and poor prognosis. In recent years, the rapid and great advances in targeted therapy and immunotherapy have brought them hope. Unfortunately, most patients develop acquired resistance after treatment. Therefore, it is urgent to explore the molecular mechanisms of acquired resistance in NSCLC. A study disclosed the crucial role of circIGF2BP3 in modulating antitumor immunity in NSCLC. Specifically, YTHDC1 elevated the biogenesis of m6A-modified circIGF2BP3, which could bind to miR-328-3p and miR-3173-5p to promote the expression of PKP3, which further increased PD-L1 abundance by enhancing PD-L1 deubiquitination mediated by OTUB1, thereby promoting tumor immune evasion. Further animal experiments demonstrated the negatively regulatory role of circIGF2BP3 in anti-PD-L1 therapy in NSCLC [195]. Since the advent of epidermal growth factor receptor tyrosine kinase inhibitors (EGFR-TKIs), including gefitinib, erlotinib and osimertinib, the overall survival rate of most patients with EGFR mutation-positive NSCLC has been substantially improved. However, many patients gradually develop resistance to EGFR-TKIs. Liu’s group revealed that hsa_circ_0005576 could elevate the mRNA level of insulin-like growth factor 1 receptor (IGF1R) by sponging miR-512-5p in osimertinib-resistant LUAD cells. IGF1R has been demonstrated to promote proliferation and anti-apoptosis through PI3K/AKT and MAPK/ERK1/2 signaling activation, resulting in osimertinib resistance in LUAD [186]. Besides, ASK1/JNK/p38 signaling pathway is tightly related to apoptosis [196, 197]. CircASK1, originated from the ASK1 gene, enabled to encode ASK1-272aa in LUAD. This novel protein interacted with Akt1 to impede Akt1-medidated ASK1 phosphorylation, and then potentiated apoptosis induced by ASK1/JNK/p38 signaling pathway. Noteworthily, YTHDF2 was found to increase the degradation of m6A-modified circASK1, thereby repressing apoptosis and further contributing to gefitinib resistance [51].

### CircRNAs affect m6A modification in cancer therapeutic resistance

**Fig. 6:**
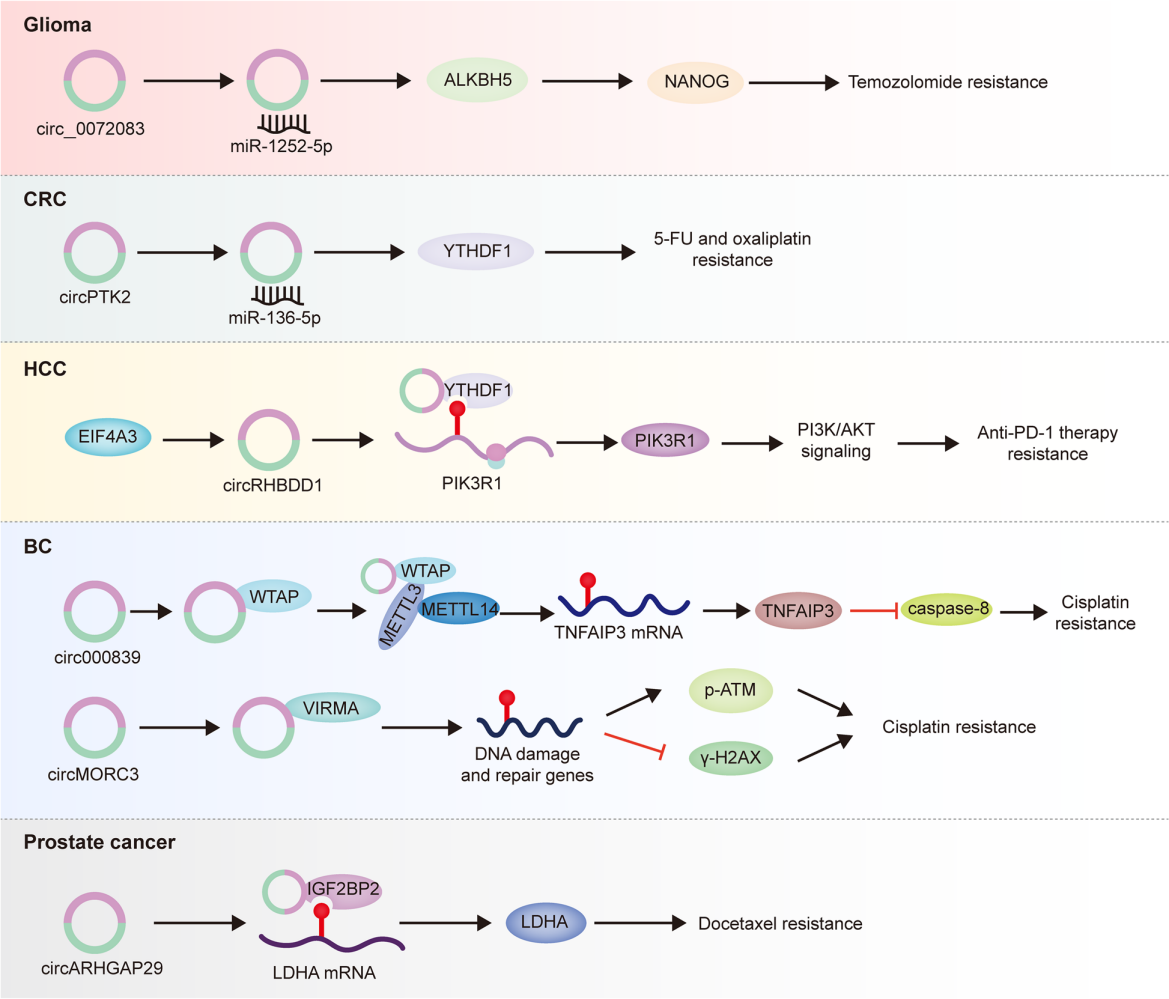
CircRNAs affect m6A modification in cancer therapeutic resistance. CircRNAs modulate the expression of or interact with m6A regulators, thereby influencing m6A functions in multiple cancers. (1) In glioma: circ_0072083 promoted ALKBH5 expression, leading to temozolomide resistance. (2) In CRC: circPTK2 elevated YTHDF1 level, resulting in 5-FU and oxaliplatin resistance. (3) In HCC: circRHBDD1 recruited YTHDF1, leading to anti-PD-1 therapy resistance. (4) In BC: circ0008399 facilitated the formation of MTC through combining with WTAP, thereby elevating cisplatin resistance. Furthermore, circMORC3 promoted cisplatin resistance by interacting with VIRMA. (5) In prostate cancer: circARHGAP29 interacted with IGF2BP2, thus augmenting docetaxel resistance

As described above, m6A modification exerts a regulatory role in circRNAs. More noteworthily, abnormalities in circRNAs expression can take effects on m6A modification in cancer. In the past 5 years, these studies on m6A regulated by circRNAs have been accumulating, mainly focusing on CRC, HCC, BC, glioma and prostate cancer (Table 4, Fig. 6).

**Table 4:** CircRNAs affect m6A modification in cancer therapeutic resistance

Cancer types	CircRNAs	Roles of circRNAs in m6A	Functions	Mechanisms	Ref.
Glioma	circ_0072083	Promotes ALKBH5 expression via sponging miR-1252-5p	Temozolomide resistance	Maintains glioma stem cells	[198]
CRC	circPTK2	Elevates YTHDF1 level by targeting miR-136-5p	5-FU/oxaliplatin resistance	–	[199]
HCC	circRHBDD1	Recruits YTHDF1	Anti-PD-1 therapy resistance	Elevates glycolysis	[200]
BC	circ0008399	Facilitates the formation of MTC through combining with WTAP	Cisplatin resistance	Boosts anti-apoptosis	[201]
	circMORC3	Interacts with VIRMA and elevates global m6A level	Cisplatin resistance	Promotes DNA repair and suppresses DNA damage	[202]
Prostate cancer	circARHGAP29	Interacts with IGF2BP2	Docetaxel resistance	Promotes glycolysis	[156]

#### Glioma

Glioma is the most prevalent class of primary central nervous system tumor with high recurrence and poor prognosis [203]. It was reported that the presence of glioma stem cells is a vital reason for high recurrence and mortality [204]. Recently, the m6A demethylase ALKBH5 was discovered to facilitate invasion and radioresistance of glioblastoma stem cells by downregulating the expression of HRR-related genes including CHK1 and RAD51 [205]. ALKBH5 is a promising target for surmounting radioresistance and reducing recurrence of glioblastoma. Moreover, ALKBH5 also affects the therapeutic effect of the first-line chemotherapy drug temozolomide in glioma. As found by Ding et al., upregulated ALKBH5 augmented demethylation of NANOG to increase its expression in temozolomide-resistant glioma cells. More strikingly, the upregulation of ALKBH5 was attributed to circ_0072083 sponging to miR-1252-5p [206]. NANOG, one of the pluripotency factors, is of importance to maintain CSCs in cancer [207]. In short, circ_0072083 depletion inhibited ALKBH5 expression via releasing miR-1252-5p, which further downregulated NANOG expression, thus alleviating temozolomide resistance by inhibiting the maintenance of glioma stem cells.

#### Colorectal cancer (CRC)

CRC is one of the common tumors of the digestive system [198]. Since the incidence and mortality of CRC are growing year by year worldwide, it is necessary to identify effective diagnostic and prognostic biomarkers in CRC. As an m6A reader, YTHDF1 is responsible for RNA translation in eukaryotes, and has been demonstrated to have significant functions in CRC [208–210]. For instance, Chen et al. confirmed the upregulation of YTHDF1 in cisplatin-resistant CRC cells, and silencing YTHDF1 alleviated cisplatin resistance via dampening GLS-mediated glutamine metabolism in CRC [209]. But they haven’t investigated the mechanisms in the upregulation of YTHDF1. One research elucidated that c-Myc contributed to YTHDF1 expression in CRC. YTHDF1 deficiency had an inhibitory effect on CRC proliferation and chemosensitivity in vivo and in vitro [210]. Additionally, circRNAs were found to be associated with YTHDF1 expression. As clarified by Jiang et al., circPTK2, serving as a miRNA sponge, elevated YTHDF1 level by targeting miR-136-5p, thereby promoting CRC progression, 5-FU and oxaliplatin tolerance [211]. However, their research didn’t reveal the downstream mechanisms of YTHDF1 in CRC. More efforts are needed to unveil the function of YTHDF1 in CRC progression and therapeutic resistance.

#### Hepatocellular carcinoma (HCC)

With the accumulation of epigenetic studies, it has been gradually realized that aberrant epigenetic modifications of RNA, especially m6A modification may result in HCC occurrence, progression and even therapeutic resistance [190]. YTHDF1 belongs to the YTH domain family, which is responsible for the modulation of RNA translation. Attractively, some researchers demonstrated that several circRNAs could affect the expression and functions of YTHDF1 in HCC. A compelling analysis based on TCGA and GEO database revealed that circMAP2K4 targeted hsa-miR-139-5p to elevate YTHDF1 level, and contributed to HCC cell proliferation, but the potential functions of YTHDF1 remain elusive [56]. Furthermore, circRHBDD1 was uncovered to be substantially increased in HCC tissues, owing to EIF4A3-induced cyclization of circRHBDD1. The highly expressed circRHBDD1 aided the translation of PIK3R1 via recruiting YTHDF1, which potentiated PI3K/AKT signaling that was highly relevant to glycolysis, thereby restricting the efficacy of anti-PD-1 therapy in HCC [199]. Accordingly, circRHBDD1 is expected to be a novel therapeutic target for improving immunotherapeutic outcome in HCC.

#### Bladder cancer (BC)

BC originates from urinary system, and its incidence ranks 10th in the world [1]. With the development of acquired resistance, its drug-resistant recurrence has become a serious problem in clinical treatment. Mounting studies delineated that circRNAs exerted regulatory roles in BC by interacting with some m6A regulators to affect mRNA expression. More recently, circPTPRA was corroborated to suppress BC progression by interacting with IGF2BP1 to prevent recognition of m6A-modified MYC and FSCN1, thereby limiting their expression [58]. The assembly of MTC is of great significance for catalyzing m6A modification formation in RNAs. Circ0008399 facilitated the formation of MTC through combining with WTAP, thereby improving the abundance of m6A residues on TNFAIP3. Moreover, m6A-modificated TNFAIP3 expression was further upregulated to boost anti-apoptosis, thus inhibiting the therapeutic efficiency of cisplatin in BC [200]. DNA damage repair is a vital mechanism of resistance in various tumors [66, 99, 141]. Su et al. found that circMORC3 could elevate global m6A level and interact with VIRMA in BC. Besides, overexpressed circMORC3 increased p-ATM and decreased γ-H2AX expression [212]. Collectively, we speculate that circMORC3 may bind to VIRMA to augment the formation of MTC, thereby further upregulating m6A level of DNA damage response genes, leading to cisplatin tolerance in BC.

#### Prostate cancer

Prostate cancer is one of the frequently diagnosed cancers in men [202]. In 2014, docetaxel was approved by FDA as first-line chemotherapy drug for metastatic prostate cancer [213]. However, it is a pity that the effectiveness of docetaxel is discounted due to acquired resistance in prostate cancer. Aerobic glycolysis is strongly relevant to tumor growth and therapeutic resistance. For example, the loss of miR-103a-3p increased TRIM66 expression, which was instrumental to glycolysis and docetaxel resistance in prostate cancer [214]. Furthermore, LDHA is responsible for the production of lactate from pyruvate, indicating its critical function in aerobic glycolysis. Interestingly, Jiang and colleagues unveiled that EIF4A3-driven circARHGAP29 enabled to elevate the level of LDHA in docetaxel-resistant prostate cancer. In the term of mechanism, circARHGAP29 promoted LDHA-mediated glycolysis by interacting with m6A reader IGF2BP2 to stabilize LDHA expression, which disclosed the key role of circARHGAP29 in docetaxel-related chemoresistance [156].

## Conclusions and future prospects

The development of therapeutic resistance has been serious obstacles to cancer treatment, resulting in high mortality and poor prognosis in cancer patients. Hence, it is of great significance to comprehensively explore the possible molecular mechanisms of cancer therapeutic resistance. Although numerous studies have shown that many factors may affect the sensitivity of tumor cells to therapy, the underlying molecular mechanisms of therapeutic resistance have not been fully clarified. In the current review, we summarized the latest advances of mutual regulation between m6A modification and circRNAs in cancer, as well as their impacts on therapeutic resistance, which provides promising insights and future directions in reversal of therapeutic resistance in cancer.

Epigenetic regulation, especially alterations in RNA modification, can affect post-transcriptional gene expression in a variety of diseases, including cancer. We summarized the abnormal alterations of a common RNA modification, m6A modification, in tumor cells that can result in dysregulation of gene expression, such as ABC transporter gene family, OATP transporter genes, DNA damage and repair genes, autophagy-inducing genes, anti-apoptotic and pro-apoptotic genes, which contribute to cancer recurrence and therapeutic resistance. Besides, whether dysregulation of circRNAs in therapy-resistant tumors is modulated by these epigenetic mechanisms remain largely unknown. In this review, we find that m6A modifications are widely exist in circRNAs, and participate in the regulation of their biogenesis, localization, degradation and translation, which may lead to abnormal expression of circRNAs. But only several m6A-modified circRNAs are discovered to be related to the response to cancer treatment. Research on the impacts of m6A-modified circRNAs on cancer therapeutic resistance still remains at the infancy stage.

Identifying m6A motifs on circRNAs is one of the technical challenges. In current decades, growing number of m6A detection technologies have been developed, such as methylated RNA immunoprecipitation sequencing (MeRIP-seq), methylation-iCLIP (miCLIP), m6A labeling-based sequencing (m6A-label-seq), and diversity arrays technology sequencing (DART-seq), etc. However, these methods have limitations and remain to be developed. For instance, MeRIP-seq is an antibody-dependent method that may lead to false-positive signals due to non-specificity of antibodies. Furthermore, m6A-label-seq can identify m6A sites with single-base resolution, but only a limited number of m6A residues can be detected [215–218]. Therefore, it is needed to develop more accurate and effective methods to detect m6A levels on circRNAs, and to identify important m6A-modified circRNAs in resistant tumor tissues and cells. Moreover, extensive evidence indicates that other RNA modifications, such as 5-methylcytosine (m5C) and pseudouridine (Ψ), are widespread in ncRNAs including lncRNAs, miRNAs and ribosomal RNAs [219]. Hence, we speculate that circRNAs levels are not limited to be affected by m6A modification, but may also be regulated by other RNA modifications. What roles they might play in the regulation of circRNAs is an interesting topic worth studying, and more efficient detection of other types of modified-circRNAs also relies on the development of detection technologies.

Recently, researchers have disclosed numerous natural products and lead compounds targeting m6A writers/readers/erasers. For instance, SUN et al. reported that Saikosaponin D (SsD) could elevate global m6A modification level via inhibiting m6A demethylase FTO, which further relieved leukemia resistance to TKIs treatment by decreasing the stability of MTHFR and BCL-2 [201]. In addition to SsD, there are some other modulators targeting m6A regulatory proteins, such as curcumin, fusaric acid, chidamide and STM2457 [220]. The finding of these modulators targeting m6A may provide novel and effective strategies for overcoming therapeutic tolerance in cancer.

As a multifunctional and distinctive ncRNA, circRNAs have been demonstrated to be tightly relevant to therapeutic resistance in various cancers, and are expected to be promising biomarkers and targets [4]. We summarized that circRNAs modulate therapeutic sensitivity by affecting drug efflux, DNA damage repair, apoptosis, TME, autophagy, EMT, CSCs and glycolysis. Except these mechanisms, other possibilities, for instance, the modulation of drug influx by circRNAs have not been reported, which merits further investigation.

In current years, mounting studies have uncovered that circRNAs are abundant and stable in exosomes. In addition, exosomes have been found to promote cancer progression by serving as trucks to transport cargo molecules including circRNAs, and are widely distributed in body fluids such as plasma, saliva and urine [221]. Therefore, it cannot be ignored that exosomal circRNAs may act as ideal markers for cancer diagnosis and prognosis in liquid biopsy, and highly accurate detection techniques are instrumental to achieve this idea in the future. Strikingly, Chen et al. disclosed that exogenous circRNAs could induce innate immunity through activating retinoic acid-inducible gene-I (RIG-I) pathway, implying that exogenous circRNAs may function as a novel tumor antigen to induce antitumor immunity in vivo [222]. Furthermore, some circRNAs were found to be translated via internal ribosome entry site (IRES) and m6A-dependent pathways. Thus, developing circRNA vaccines based on their coding abilities may provide a beneficial direction for future antitumor immunotherapy and reversal of therapeutic resistance [223].

Besides, some circRNAs can modulate the expression of m6A regulators based on the function of sponging miRNAs, or impacting mRNA m6A modification process via interacting with m6A writers/readers/erasers in cancer. Whether circRNAs can affect m6A modification based on other biological functions, such as regulating gene transcription or being translated, is unknown.

In short, m6A modification, circRNAs and their interactions have exhibited momentous roles in modulating cancer therapeutic resistance. In the future, drug development targeting m6A and circRNAs might be beneficial in overcoming resistance and provide new therapeutic opportunities for cancer patients.

## Data Availability

Not applicable.

## Abbreviations

TME: tumor microenvironment
EMT: epithelial-mesenchymal transition
ncRNAs: non-coding RNAs
miRNAs: microRNAs
lncRNAs: long non-coding RNAs
circRNAs: circular RNAs
SMOC2: secreted modular calcium-binding protein 2
PC: pancreatic cancer
m6A: N6-methyladenosine
ABC: ATP-binding cassette
N: nitrogen
A: adenylate
MTC: methyltransferase complex
METTL3/14/16: methyltransferase-like 3/14/16
WTAP: Wilm’s tumor 1-associated protein
ZC3H13: zinc finger CCCH-type containing 13
VIRMA: vir-like m6A methyltransferase associated
RBM15/15B: RNA-binding motif protein 15/15B
FTO: fat mass and obesity-associated protein
ALKBH5: alkB homolog 5
eIF3: eukaryotic initiation factor 3
ceRNAs: competing endogenous RNAs
pre-mRNAs: precursor mRNAs
RBPs: RNA-binding proteins
BHB: bulge-helix-bulge
MREs: miRNA response elements
3′ UTRs: 3′ untranslated regions
IRES: internal ribosome entry site
HCC: hepatocellular carcinoma
TMD: transmembrane domain
ATP: adenosine triphosphate
IGF2BP3: Sulin-like growth factor 2 mRNA-binding protein 3
CRC: colorectal cancer
ERRγ: estrogen receptor related receptors
NSCLC: non-small cell lung cancer
OATP: organic anion transporting polypeptides
ccRCC: clear cell renal cell carcinoma
HNF3γ: hepatocyte nuclear factor 3γ
ULK1: unc-51-like kinase 1
GC: gastric cancer
NER: nucleotide excision repair
BER: base excision repair
HRR: homologous recombination repair
NHEJ: non-homologous end joining
ERCC1: excision repair cross-complementation group-1
CSCC: cervical squamous cell carcinoma
DSBs: DNA double strand breaks
EMP3: epithelial membrane protein 3
LUAD: lung adenocarcinoma
Tregs: T regulatory cells
MDSCs: myeloid-derived suppressor cells
TAMs: tumor-associated macrophages
TGF-β: transforming growth factor β
ICC: intrahepatic cholangiocarcinoma
HNSCC: head and neck squamous cell carcinoma
CSCs: cancer stem cells
CC: colon cancer
ESCC: esophageal squamous cell carcinoma
5-FU: 5-fluorouracil
BCL-2: B-cell lymphoma-2
IAPs: inhibitors of apoptosis proteins
TKIs: tyrosine kinase inhibitors
CK2α: casein kinase 2 α
BC: bladder cancer
GBM: glioblastoma multiforme
EC: esophageal cancer
ECM: endothelial monolayer permeability
CML: chronic myeloid leukemia
ESRP1: epithelial splicing regulatory protein 1
LDHA: lactic dehydrogenase A
HKs: hexokinases
HSCC: hypopharyngeal squamous cell carcinoma
IGF1R: nsulin-like growth factor 1 receptor
m5C: 5-methylcytosine
Ψ: pseudouridine
SsD: Saikosaponin D
MeRIP-seq: methylated RNA immunoprecipitation sequencing
miCLIP: methylation-iCLIP
m6A-label-seq: m6A labeling-based sequencing
DART-seq: diversity arrays technology sequencing
